# Prevalence and Factors Associated with Falls among Older Outpatients

**DOI:** 10.3390/ijerph18084041

**Published:** 2021-04-12

**Authors:** Van-Anh Thi Ha, Tam Ngoc Nguyen, Thanh Xuan Nguyen, Huong Thi Thu Nguyen, Thu Thi Hoai Nguyen, Anh Trung Nguyen, Thang Pham, Huyen Thi Thanh Vu

**Affiliations:** 1 Outpatient Department, National Geriatric Hospital, Hanoi 100000, Vietnam; chatonvananh@yahoo.com; 2 Department of Geriatrics, Hanoi Medical University, Hanoi 100000, Vietnam; ngoctam@hmu.edu.vn (T.N.N.); xuanthanh1901vlk@gmail.com (T.X.N.); thuhuonglk@hmu.edu.vn (H.T.T.N.); nththu.bvlk@gmail.com (T.T.H.N.); trunganhvlk@gmail.com (A.T.N.); phamthang@hmu.edu.vn (T.P.); 3 Scientific Research Department, National Geriatric Hospital, Hanoi 100000, Vietnam; 4 Dinh Tien Hoang Institute of Medicine, Hanoi 100000, Vietnam

**Keywords:** falls, elderly, outpatients, geriatric syndromes, associated factors

## Abstract

Falls in older people are a major public health issue, as they are associated with increased risks of morbidity and mortality. This study aims to investigate the prevalence and factors associated with falls among older outpatients. A cross-sectional study was conducted in 539 outpatients aged 60 and over at the National Geriatric Hospital, Hanoi, Vietnam. Falls and their associated factors were analyzed by multivariable logistic regression. The prevalence of falls was 23.7% (single fall 17.9%, recurrent falls 5.8%). The majority of falls occurred at home (69.6%) and were caused by a slippery floor (51.6%). After falling, most patients sustained physical injuries (65.6%); notably, women suffered more severe injuries than men. Alcohol consumption, using psychotropic medications, having three or more comorbidities, hypertension, COPD, urinary incontinence, frailty, fear of falling, ADL/IADL limitation, slow walking speed and mobility impairment were significantly associated with falls. Overall, the data indicated that falls were prevalent among older outpatients. Behavior factors, comorbidities, geriatric syndromes and physical function were substantially associated with falls, suggesting that most falls are preventable. Further longitudinal studies of longer periods are needed to comprehensively investigate the risk factors for falls.

## 1. Introduction

Aging is an irreversible natural phenomenon of global concern. Falls in the elderly are among the most serious public health problems in this era due to the considerable increase in fall-related morbidity and mortality in the aging population [1,2,3]. With an increasingly active and expanding older population worldwide, the burden of falls and fall-related consequences is expected to rise in the future. The World Health Organization defines falls as “inadvertently coming to rest on the ground, floor or other lower level, excluding intentional change in position to rest in furniture, wall or other objects” [4]. The reported rate of falls among older people in recent studies worldwide ranges from 4% to 35% and increases steadily with age [5,6,7,8,9,10,11,12].

Like many high- and middle-income countries, Vietnam is faced with an aging population, which leads to an increase in the prevalence of diseases and puts society and the healthcare system under strain. An increasing rate of falls among senior citizens has been associated with rising injury cases, causing hospitalization, disability and even premature death. Elderly individuals who suffer from multiple accompanying diseases are more likely to sustain serious damage during a fall [13]. The cost of falls, including the cost of ambulance services and medical treatment, is exorbitant.

The increase in falls and fall-related injuries stemming from the increasing proportion of the population being elderly presents challenges to the community and must be tackled. However, due to their multifactorial etiology, preventing falls is not simple or straightforward. Recent studies have identified several factors associated with falls among the elderly, including environmental and sociodemographic factors and biological and behavioral factors [4,9]. The rate of falls increases when multiple risk factors are combined: The prevalence of falls increases from 8% without risk factors to up to 78% with 4 risk factors [9]. It is imperative to develop effective methods to identify factors that predispose individuals to falls in specific older populations to reduce modifiable risk factors for falls among older adults. Nevertheless, most studies have been conducted in the community or in nursing homes; thus, almost all older populations who receive medical check-ups and treatment in outpatient clinics are not evaluated for fall risk frequently, especially in Vietnam. The present study aimed to assess the characteristics of and factors associated with falls among participants in outpatient clinics in Hanoi, Vietnam.

## 2. Materials and Methods

### 2.1. Study Design and Participants

This cross-sectional study was conducted in consecutive patients aged 60 or older who visited the outpatient department at the National Geriatric Hospital, Hanoi, Vietnam, from January 2018 to December 2018. Participants were excluded if they: (1) were unable to understand and/or answer the questions and perform the gait speed and Timed Up and Go tests, (2) suffered from acute or severe illness, (3) had cognitive impairment (based on the Montreal cognitive assessment (MoCa) [14]), or (4) did not agree to participate in the study. The study was approved by the National Geriatric Hospital Ethics Committee, Vietnam, and written consent was obtained from all participants.

### 2.2. Sample Size Calculation

The sample size was calculated using a single population proportion formula, sample size: n = Z2 1 − α/2 × (*p* × (1 − *p*)/d2), with n = the required sample size, Z1 − α/2 = 1.96 (with α = 0.05 and 95% confidence interval), *p* = 0.279 (the prevalence of falls among older outpatients according to the 2007 study by Moreira et al. [15]), and d = precision (assumed to be 0.04). Therefore, our study’s sample size was calculated to be at least 483 participants, and with an allowable margin of error of 5%, the required sample size was determined to be at least 507 participants.

### 2.3. Data Collection

The face-to-face interviews were conducted by six physicians working at the National Geriatric Hospital and Hanoi Medical University. All physicians had both work and research experience in the field of geriatrics. Before conducting data collection, the study was thoroughly explained to them (including information about objectives, subjects and the questionnaire), and they were trained in interviewing the patients.

### 2.4. Questionnaire

Each respondent was required to complete a structured questionnaire based on the face-to-face interview with the following criteria:

#### 2.4.1. Falls

Participants were asked if they had experienced falls in the past year (one or more falls). Those resulting from outside events, such as motor vehicle accidents or violence, were excluded. Those who had fallen were also asked about the number and location of falls (bedroom, bathroom, on a stair, hallway, outside the home, other), fall circumstances (dizziness when standing up, slippery floor, loss of balance, tripping/thrust, riding a bike/motorbike, other) and health problems after falling (having any injury, fracture, open wound/bruise, immobility, hospitalization after falls).

#### 2.4.2. Independent Variables

Sociodemographic variables comprised sex (men and women), age (divided into two groups: 60–79 and ≥80), marital status (single, widowed and married), area of residence (countryside, city), economic status (poor and non-poor, based on participants’ self-report) and education level (pre-high school, high school, and tertiary education).

Anthropometric measurement: body mass index (BMI) was calculated using the formula weight/height^2^ (kg/m^2^) and was categorized into four groups: underweight (<18.50), normal (18.50–22.99), overweight (23–24.99) and obesity (≥25) [16].

Behavioral factor: Alcohol intake was self-reported and classified as alcoholic consumption versus non-alcoholic consumption.

Participants were also categorized according to their use of psychotropic medications (hypnotics or sedatives, narcotic analgesics, anticonvulsants, tricyclic antidepressants and selective serotonin reuptake inhibitors) according to the Iowa Drug Information System Coding System) [17].

Comorbidities included the number of diagnosed comorbidities (<3 vs. ≥3), which included hypertension, diabetes, chronic obstructive pulmonary disease (COPD), peripheral vascular disease and history of stroke. Comorbidities were obtained from medical records based on doctors’ diagnoses.

Depressive symptoms were screened using a 15-item geriatric depression scale (GDS). On this scale, the maximum score is 15 points, and a total score ≥ of 5 was considered to indicate depression [18].

Urinary incontinence: Participants were asked about symptoms of urinary incontinence by answering the 3 incontinence questions (3IQ) without assistance [19]. Responses were dichotomous (yes or no).

Frailty status was evaluated using the Fried phenotype with five criteria: (1) unintentional weight loss of ≥5% or ≥4.5 kg in the last year; (2) weakness (obtained using handgrip strength measurements); (3) low energy (exhaustion: obtained by asking patients two questions from the Centre for Epidemiologic Studies depression scale (CES-D)); (4) slowness (assessed based on slow walking speed using the 4-m walk test) and (5) low physical activity (assessed through participant self-report). Participants with three or more of these criteria were classified as having frailty syndrome [20].

Fear of falling: The fall efficacy scale—international (FES-I) was created to assess the fear of falling. A total score of >23 indicates a sense of fear when falling [21].

Functional ability: The Katz and Lawton scales were used to evaluate individuals’ functional ability in the performance of activities of daily living with or without instruments (IADL and ADL, respectively). For the ADL scale, the maximum score is 6, with a total score of <6 representing a decrease in daily functional activities [22]. For the IADL, the maximum score is 8, with a total score <8 indicating a decline in daily functional activities with an instrument [23].

Quality of sleep was assessed using the Pittsburgh sleep quality index (PSQI). The PSQI is a self-rating scale that distinguishes “poor” from “good” sleep by measuring seven domains: all subjective, sleep latency, sleep duration, sleep efficiency, sleep quality, use of sleeping medication and daytime dysfunction over the last month. Each domain is scored from 0 (good sleep quality) to 3 (poor sleep quality), and the total score ranges from 0 to 21. A total score ≥of 5 indicates poor sleep quality [24].

#### 2.4.3. Gait Speed Test

The 4 meter walking test (with 2 m provided for acceleration/deceleration) was used to measure gait speed (m/s). The results were divided into normal and slow walking speeds, with gait speeds of ≥0.9 m/s or <0.9 m/s, respectively [25].

#### 2.4.4. Mobility Assessment Test

The timed up and go test (TUG) was used to assess balance, walking ability and functional mobility in elderly populations. Those completing the test ≥13.5 s were classified into the “mobility impairment” group. A faster time indicates better functional performance, thus placing participants into the “normal” category [26].

### 2.5. Statistical Analysis

The collected data were handled using Statistical Package for Social Sciences (SPSS) version 22. Descriptive statistics were used for all participants’ characteristics and factors associated with self-reported falls. In particular, the continuous variables were examined by means and standard deviation (SD) and categorical variables were described using frequencies and percentages. Chi-squared tests were performed to compare different variables (categorical variables) between different groups. Univariate and multivariate logistic regression models were used to identify factors associated with falls in the participants, with odds ratios (ORs) and 95% confidence intervals (CIs) calculated for all models. Tests with *p*-values ≤of 0.05 were considered to be statistically significant.

Appropriate variables for multivariate regression models were selected using Directed acyclic graphs (DAGs). DAGs were used to construct the conceptual diagram representing causal relationships of all variables in the network [27,28]. Based on DAGs, the minimal sufficient adjustment sets (MSAS), which are sets of variables needed to block all backdoor paths (confounding paths) of the relationship between some related factors and falls, were obtained [27,28]. For each associated factor, we drew a DAG to find confounders for inclusion in the multivariate model (Supplementary File). The DAGs were constructed using the online software DAGitty, available at https://www.dagitty.net (accessed on 17 March 2021) [27].

## 3. Results

Table 1 illustrates the general characteristics of participants. Of the 539 total participants, 128 (23.7%) self-reported that they experienced a fall at least once in the previous year. The mean age of the participants was 69.4 ± 7.5 years. Notably, 28.1% of fallers were aged 80 years and over, while 8.8% of non-fallers were 80 years of age or older. Health problems like hypertension, stroke, peripheral vascular disease, COPD and geriatric problems (frailty, depressive symptoms, urinary incontinence, poor sleep quality, fear of falling, ADL limitation and IADL limitation) were predominate in the fallers. A greater proportion of the participants who suffered from more than two comorbidities and those who used psychotropic medications were seen in the faller group than in the non-faller group. The percentage of those performing poorly on the walking speed and TUG tests was remarkably higher among fallers than among non-fallers. In addition, the percentages of patients with underweight, low-income, high educational level and alcohol intake in the faller group were significantly higher than those in the non-faller group.

Table 2 shows the characteristics of falls among the participants. Ninety-seven participants had a single fall (17.9%), and 31 people had two or more falls (5.8%). The majority of falls occurred at home (69.6%), mainly in the bathroom. Moreover, a high percentage of falls were caused by a slippery floor, followed by losing balance and as a consequence of dizziness when standing up. Regarding the consequences of falls, nearly two-thirds of fallers suffered from injuries, of which the majority were bruises and grazes. The degree of trauma after falling was more serious among women than among men, which can be clearly seen in figures for fracture and immobility. Meanwhile, men’s hospitalization rate was higher than in women, though the difference was not statistically significant.

Table 3 indicates results from the univariate and multiple logistic regression for factors associated with falls, including sociodemographic variables, comorbidities, geriatric syndromes and physical measures of walking speed and mobility. Participants aged 80 years and above had a 4-fold increased risk of falling (OR 4.08; 95% CI 2.44–6.82). Marital status (single) and having a low income were also associated with an increased risk of fall.

Once the models for all co-variables were adjusted, the result showed that the following factors were significantly associated with falls: alcohol consumption, using psychotropic medications, having 3 or more comorbidities, hypertension, COPD, urinary incontinence, frailty, fear of falling, ADL/IADL limitations, slow walking speed and mobility impairment on the TUG test.

## 4. Discussion

The prevalence of falls among geriatric outpatients over 12 months was 23.7% (single fall 17.9% and multiple falls 5.8%). This result is similar to that of Subramanian et al. conducted in Indian outpatients aged >60 years [29]. However, the reported global fall rates in community-dwelling older people vary from 4% to 35% [5,6,7,8,9,10,11,12], and the proportion of recurrent falls in our study is lower than those in the studies in developed countries [30,31]. These inconsistencies could be due to differences in culture, family structure and medical care conditions across countries. A study conducted in 25 European countries revealed that the variations in falls among older citizens are partly determined by the distinctions in people’s financial backgrounds and their spending on medical care [32]. Unlike older people in developed countries, who tend to be more independent, the majority of Vietnamese elderly people live alongside their offspring and relatives, who directly take care of them [33]; these family caretakers would likely be even more cautious when their parents or grandparents fall, which may predispose them to a decreased likelihood of recurrent falls.

We observed that the majority of falls occurred indoors, primarily in the bathroom. This is in line with the results of previous studies in which the bathroom was found to be a hazardous space carrying risks of falls, such as a slippery floor, poor lighting and a shortage of handrails and non-slip mats [8,34]. Making small changes to home hazards may reduce the occurrence of falls [35,36]. Our results match those of recent studies that indicated that most fallers suffer from physical injuries and that this was experienced more seriously by women than by men [37,38,39]. This finding is understandable because osteoporosis is more prevalent in women than in men at the same age [40]. A higher rate of falls and related injuries among older outpatients in this study suggest that preventive interventions for falls in these subjects should be a top priority.

The results showed that a higher percentage of impoverished people experienced falls than individuals from other income groups. This finding is similar to that of a study conducted in 25 European countries that revealed that the variations in falls among older citizens are partly determined by the distinctions in people’s financial background and their spending on medical care [41]. Accordingly, the lack of access to healthcare resources in poor citizens increases the risk of falls. A low standard of living and lack of caregiving were included in the fall risk model [42]. Another finding in this study was that fallers were significantly older than non-fallers, which is in line with what has been reported in several studies and systematic reviews: People with higher ages have an increased risk of falls [43,44]. Since age has been associated with an elevated risk of developing diseases, age may contribute to the correlation between comorbidities and falls. Our study showed that psychotropic drug use was linked to a 3.5-fold increased risk of falls, which is in agreement with the results of the study by Johnell and Cox [45,46]. This could be due to the side effects of psychotropic drugs, which include gait disorder, balance problems and impaired reaction time and other sensorimotor functions, which result in ataxia, drowsiness, dizziness, postural disturbances and impaired motor coordination [47]. These results should thus prompt practitioners to consider this when prescribing psychotropic drugs for older patients.

In this study, alcohol consumption, comorbidities (hypertension, COPD, having three or more comorbidities), geriatric syndromes (urinary incontinence, frailty, fear of falling, ADL/IADL limitations), slow walking speed and poor performance on the TUG test demonstrated a significant association with falls. These findings are consistent with those of other studies revealing that elderly people with one or more diseases experience a higher risk of falls [31,48]. ADL/IADL limitations have been shown to impact muscle function and balance or mobility capacity, as well as social isolation, thereby increasing the incidence of falls [8,49,50]. The effect of frailty on muscular strength and functional performance was regarded as a risk factor for falls [20]. Moreover, fear of falling is viewed as an independent risk factor for reduced quality of life and falls due to its profound and detrimental effect on balance and mobility in older adults [51,52]. With regard to the control of posture and gait, slow walkers and individuals who performed poorly in the TUG had a fall risk more than four times higher than those in other groups. This was in agreement with numerous previous studies reporting that gait parameter variabilities were associated with increased fall risk, loss of independence, disability and mortality [53,54,55]. Thus, it can be assumed that the assessment of gait speed could lead to healthcare support for the elderly; by identifying a decline in gait speed, which is associated with falls, it might be possible to predict older individuals at a high risk of falls. Understanding the relevant changes related to aging is one of the most important steps in solving fall-related problems in older people.

An important strength of our study is that, to our knowledge, this is the first study to use this approach to provide insight into the prevalence of, characteristics of and factors associated with falls among older outpatients who are more prone to falls and require special consideration during treatment due to their chronic diseases. Another merit lies in a relatively large sample size from the outpatient department of a national hospital. However, there exist some limitations. First, we used a cross-sectional study design; thus, the causal relationship between the potential risk factors and falls could not be established. We also did not assess environmental factors. Moreover, this study was conducted in outpatients from a single center and, therefore, may not be generalizable to community-dwelling older adults. Further longitudinal studies over longer periods are needed to comprehensively investigate the risk factors for falls.

## 5. Conclusions

Falls were prevalent among older outpatients (23.7%), and nearly two-thirds of fallers suffered from injuries. The majority of falls happened indoors. Alcohol consumption, using psychotropic medications, hypertension, COPD, having three or more comorbidities, urinary incontinence, frailty syndrome, fear of falling, ADL/IADL limitations, slow walking speed and mobility impairment were significantly associated with an increased risk of falls. Evaluation of gait, mobility and balance, comorbidities, geriatric syndromes and medication use in older adults are needed to detect the risk of falls as well as to prevent falls in high-risk subjects. Further longitudinal studies of longer periods are needed to comprehensively investigate the risk factors for falls.

## Figures and Tables

**Table 1:** Participant general characteristics.

Characteristics		Fallers (n,%)	Non-Fallers (n,%)	Total (n,%)	*p*
All		128 (23.7%)	411 (76.3%)	539	
Mean age ($\bar{X}$ ± SD)		72.4 ± 8.4	68.4 ± 6.9	69.4 ± 7.5	<0.001
Sex	Men	73 (57.0%)	198 (48.2%)	271 (50.3%)	0.080
	Women	55 (43.0%)	213 (51.8%)	268 (49.7%)	
Age groups	60–79	92 (71.9%)	375 (91.2%)	467 (86.6%)	<0.001
	≥80	36 (28.1%)	36 (8.8%)	72 (13.4%)	
Area of residence	Countryside	57 (44.5%)	184 (44.8%)	241 (44.7%)	0.962
	City	71 (55.5%)	227 (55.2%)	298 (55.3%)	
BMI (kg/m^2^) (n = 535)	Underweight	30 (23.6%)	45 (11.0%)	75 (14.0%)	0.003
	Normal	66 (52.0%)	238 (58.3%)	304 (56.8%)	
	Overweight	16 (12.6%)	77 (18.9%)	93 (17.4%)	
	Obesity	15 (11.8%)	48 (11.8%)	63 (11.8%)	
Economic status	Poor	13 (10.2%)	5 (1.2%)	18 (3.3%)	<0.001
	Non-Poor	115 (89.8%)	406 (98.8%)	521 (96.7%)	
Education level (n = 483)	Pre-high school	48 (43.2%)	167 (44.9%)	215 (44.5%)	0.035
	High school	26 (23.4%)	122 (32.8%)	148 (30.6%)	
	Tertiary education	37 (33.3%)	83 (22.3%)	120 (24.8%)	
Marital status (n = 517)	Married	93 (73.2%)	361 (88.0%)	454 (84.5%)	<0.001
	Single/widowed	34 (26.8%)	49 (12.0%)	63 (15.4%)	
Psychotropic medications (n = 533)	Yes	35 (27.3%)	35 (8.6%)	70 (13.1%)	<0.001
	No	93 (72.4%)	370 (91.4%)	463 (86.9%)	
Alcohol intake	Yes	64 (50.0%)	136 (33.1%)	200 (37.1%)	0.001
	No	64 (50.0%)	275 (66.9%)	339 (62.9%)	
Number of diagnosed comorbidities	0	0 (0.0%)	39 (9.5%)	39 (7.2%)	<0.001
	1	37 (28.9%)	179 (43.6%)	216 (40.1%)	
	2	48 (37.5%)	139 (33.8%)	187 (34.7%)	
	≥3	43 (33.6%)	54 (13.1%)	97 (18.0%)	
Hypertension		84 (65.6%)	216 (52.6%)	300 (55.7%)	0.009
Diabetes		26 (20.3%)	65 (15.8%)	91 (16.9%)	0.236
COPD		84 (65.6%)	213 (51.8%)	297 (55.1%)	0.006
Peripheral vascular disease		13 (10.2%)	13 (3.2%)	26 (4.8%)	0.001
History of stroke		10 (7.8%)	9 (2.2%)	19 (3.5%)	0.003
Frailty (n = 514)		35 (28.9%)	15 (3.8%)	50 (9.7%)	<0.001
Depressive symtoms (n = 502)		32 (27.4%)	40 (10.4%)	72 (14.3%)	<0.001
Urinary incontinence (n = 537)		27 (21.1%)	3 (0.7%)	30 (5.6%)	<0.001
Poor sleep quality (n = 514)		105 (85.4%)	287 (73.4%)	392 (76.3%)	0.007
Fear of falling (n = 537)		86 (67.2%)	170 (41.6%)	256 (47.7%)	<0.001
ADL limitations (n = 537)		63 (49.2%)	46 (11.2%)	109 (20.3%)	<0.001
IADL limitations (n = 538)		72 (56.3%)	75 (18.3%)	147 (27.3%)	<0.001
Walking speed (n = 488)	Normal	16 (14.5%)	212 (56.1%)	228 (46.7%)	<0.001
	Slow	94 (85.5%)	166 (43.9%)	260 (53.3%)	
Mobility (n = 529)	Normal	65 (51.6%)	353 (87.6%)	418 (79.0%)	<0.001
	Impairment	61 (48.4%)	50 (12.4%)	111 (21%)	

**Table 2:** Characteristics of falls in older participants (faller group).

Characteristics		Men (n,%)	Women (n,%)	Total (n,%)	*p*
All		73 (57%)	55 (43%)	128 (23.7%)	
**Number of falls in the previous year**					
Single fall		54 (74%)	43 (78.2%)	97 (75.8%)	0.582
Recurrent falls		19 (26%)	12 (21.8%)	31 (24.2%)	
**Location of falls**					
Inside home	Bedroom	10 (13.7%)	7 (12.7%)	17 (13.3%)	0.873
	Bathroom	28 (38.4%)	17 (30.9%)	45 (35.2%)	0.382
	On stairs	16 (21.9%)	3 (5.5%)	19 (14.8%)	0.008
	Hallway	6 (8.2%)	2 (3.6%)	8 (6.3%)	0.249
Outside home		11 (15.1%)	12 (21.8%)	23 (18.0%)	0.325
Other/do not remember		11 (15.1%)	21 (38.2%)	32 (25.0%)	0.003
**Fall situations**					
Dizziness when standing up		16 (21.9%)	12 (21.8%)	28 (21.9%)	0.989
Slippery floor		40 (54.8%)	26 (47.3%)	66 (51.6%)	0.399
Loss of balance		21 (28.8%)	14 (25.5%)	35 (27.3%)	0.677
Tripping/thrust		2 (2.7%)	2 (3.6%)	4 (3.1%)	0.577
Riding a bike/motorbike		5 (6.8%)	12 (21.8%)	17 (13.3%)	0.013
Others		6 (8.2%)	3 (5.5%)	9 (7.0%)	0.405
**Any physical injury**		52 (71.2%)	32 (58.2%)	84 (65.6%)	0.051
**Type of injury**					
Fracture		5 (8.9%)	8 (22.2%)	13 (14.1%)	0.076
Bruise/grazes		46 (82.1%)	22 (61.1%)	68 (73.9%)	
Others		5 (8.9%)	6 (16.7%)	11 (12.0%)	
**Immobility after falls**		2 (4.0%)	3 (10.0%)	5 (6.3%)	0.270
**Hospitalized after falls**		23 (31.5%)	13 (23.6%)	36 (28.1%)	0.327

**Table 3:** Factors associated with fall from the univariate and multiple logistic regression.

Variables	Crude Odds Ratio ^1^ (95% CI)		Adjusted Odds Ratio ^2^ (95% CI)	
	OR	95% CI	OR	95% CI
Sex: women (men)	0.70	0.47–1.04	-	-
Age: ≥80 (60–79)	4.08 *	2.44–6.82	-	-
Marital status: single (has partner)	2.70 *	1.65–4.41	-	-
Economic status: poor (Not poor)	9.18 *	3.21–26.28	-	-
Education level: ≥high school (pre-high school)	1.07	0.70–1.64	-	-
Alcohol intake: Yes (No)	2.02 *	1.35–3.03	2.18 *	1.38–3.45
Psychotropic medications: Yes (No)	3.98 *	2.36–6.70	3.52 *	1.96–6.30
Comorbidities: ≥3 Yes (No)	3.34 *	2.10–5.33	2.87 *	1.64–5.03
Hypertension: Yes (No)	1.72 *	1.14–2.60	1.92 *	1.19–3.09
Diabetes: Yes (No)	1.36	0.81–2.25	1.41	0.78–22.58
Peripheral vascular disease: Yes (No)	3.46 *	1.56–7.67	2.16	0.86–5.41
History of stroke: Yes (No)	3.79 *	1.50–9.53	2.53	0.89–7.15
COPD: Yes (No)	1.77 *	1.17–2.68	1.60 *	1.00–2.57
Frailty: Yes (No)	10.26 *	5.36–19.62	4.85 *	2.10–11.15
Urinary incontinence: Yes (No)	36.18 *	19.77–121.63	20.02 *	5.09–78.60
Poor sleeping quality: Yes (No)	2.11 *	1.22–3.66	1.62	0.90–2.91
Depressive symtoms: Yes (No)	3.25 *	1.93–5.47	0.70	0.30–1.64
Fear of falling: Yes (No)	2.88 *	1.90–4.37	1.96 *	1.13–3.43
ADL limitations: Yes (No)	7.64 *	4.81–12.15	4.50 *	1.98–10.24
IADL limitations: Yes (No)	5.74 *	3.74–8.83	2.57 *	1.21–5.47
Walking speed: Slow (Normal)	7.50 *	4.25–13.23	4.56 *	2.46–8.43
Mobiblity: Impairment (Normal)	6.63 *	4.19–10.47	4.97 *	2.66–9.31

^1^ univariate logistic regression; ^2^ multiple logistic regression; * *p* < 0.05.

## Data Availability

The datasets of this study are available from the corresponding author on reasonable request.

## Supplementary Materials

The following are available online at https://www.mdpi.com/article/10.3390/ijerph18084041/s1, Directed Acyclic Graphs.
