# Supplementary material for: Prevalence and Factors Associated with Falls among Older Outpatients
Source: Int J Environ Res Public Health. 2021 Apr 12;18(8):4041. doi: 10.3390/ijerph18084041 (PMC8070134; doi:10.3390/ijerph18084041)
Supplement: Supplementary file 1 [file ijerph-18-04041-s001.pdf]

Supplement: Directed Acyclic Graphs

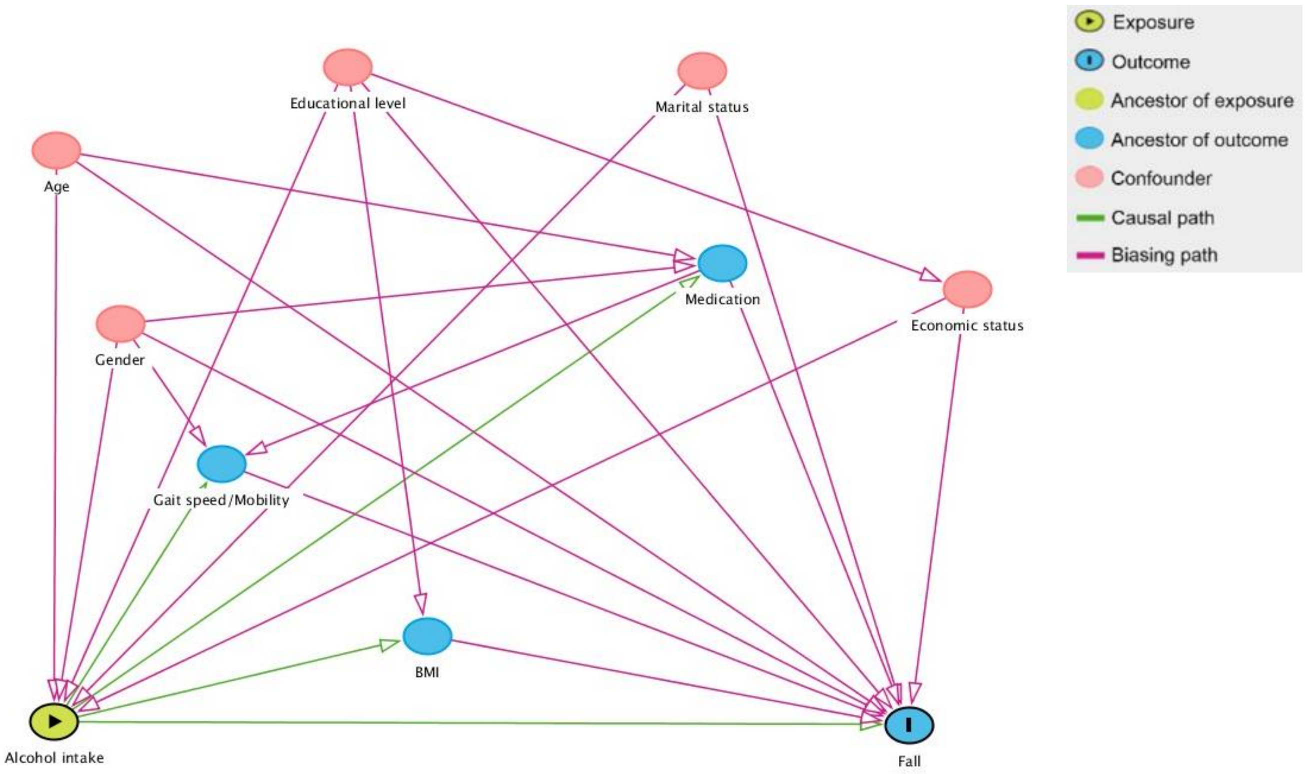

Fig 1S. Directed Acyclic Graphs for causal inference framework of alcohol intake to fall among older outpatient. Minimal Sufficient Adjustment Sets: age, gender, educational level, marital status and economic status. The graph was drawn from https://www.dagitty.net

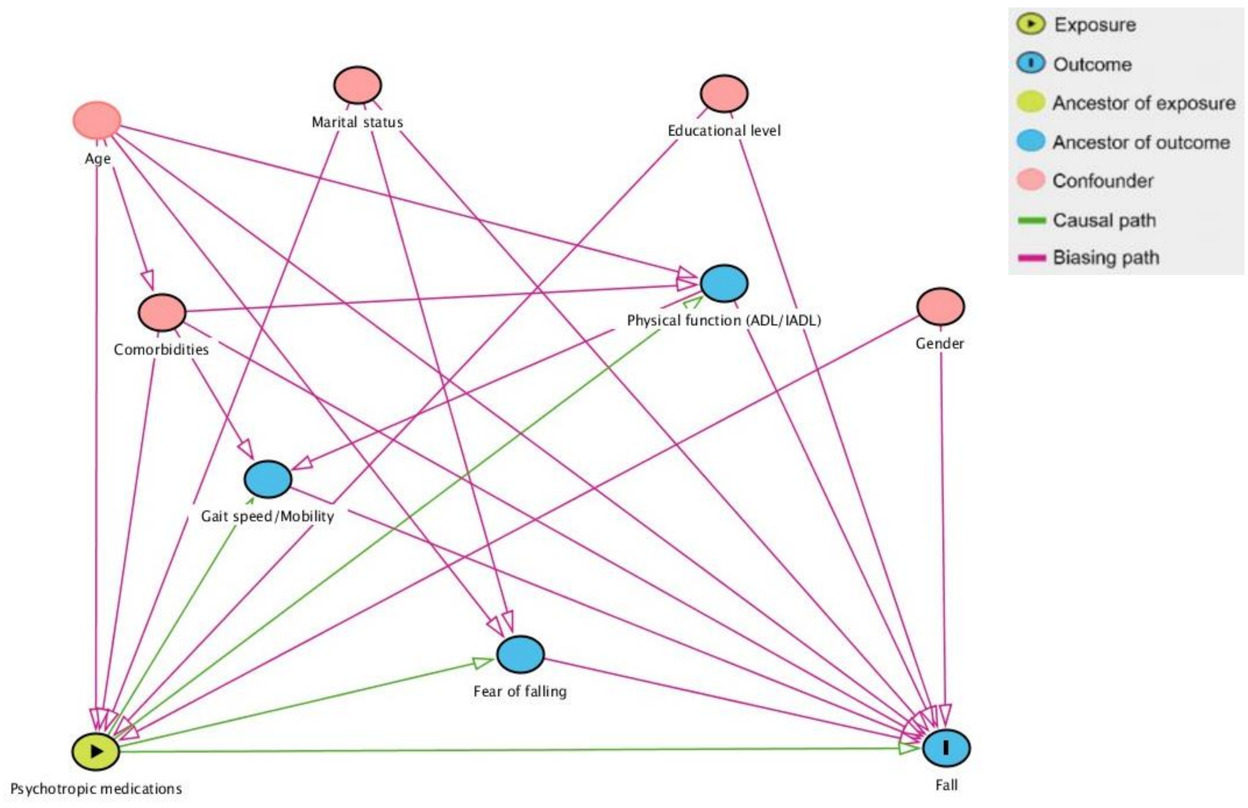

Fig 2S. Directed Acyclic Graphs for causal inference framework of psychotropic medications to fall among older outpatient. Minimal Sufficient Adjustment Sets: age, gender, educational level, marital status and comorbidities. The graph was drawn from https://www.dagitty.net

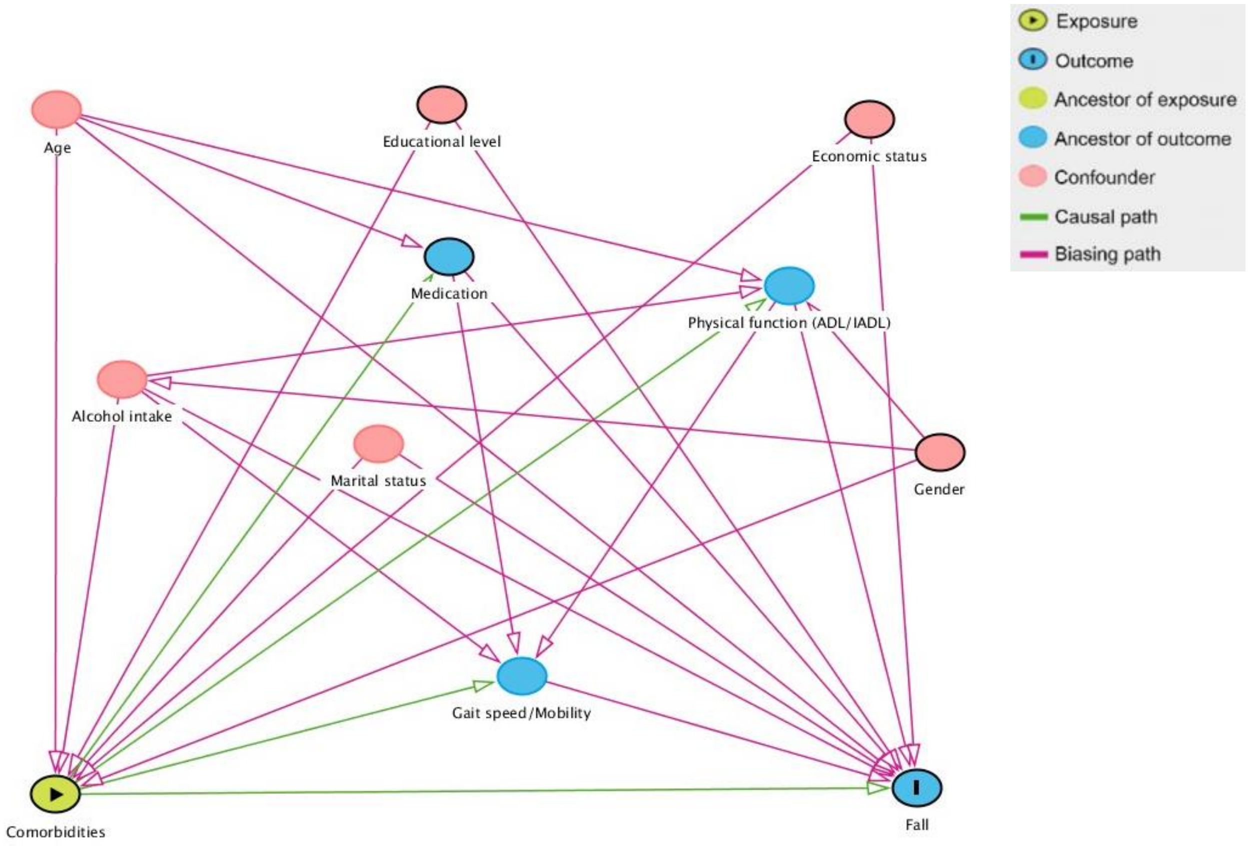

Fig 3S. Directed Acyclic Graphs for causal inference framework of comorbidities to fall among older outpatient. Minimal Sufficient Adjustment Sets: age, gender, educational level, marital status, economic status and alcohol intake. The graph was drawn from https://www.dagitty.net

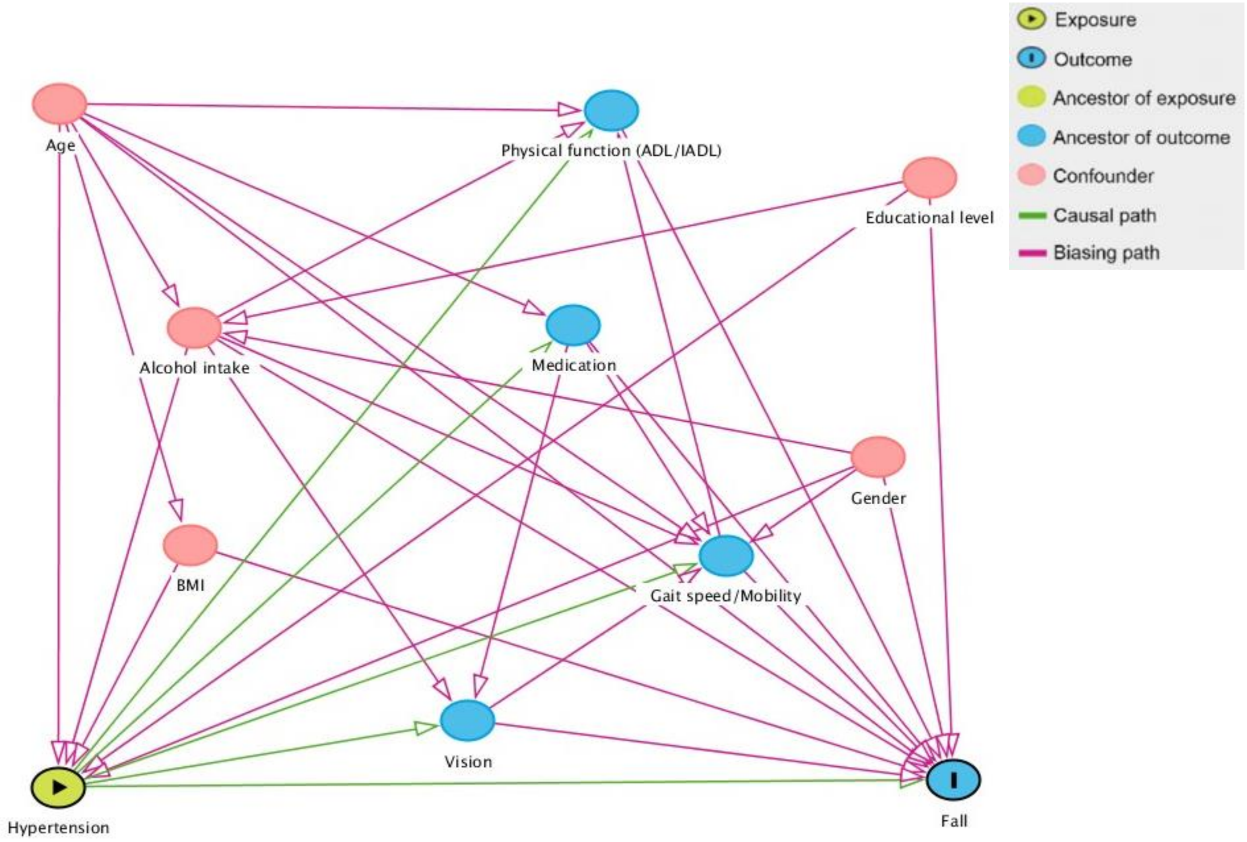

Fig 4S. Directed Acyclic Graphs for causal inference framework of hypertension to fall among older outpatient. Minimal Sufficient Adjustment Sets: age, gender, educational level, BMI and alcohol intake. The graph was drawn from https://www.dagitty.net

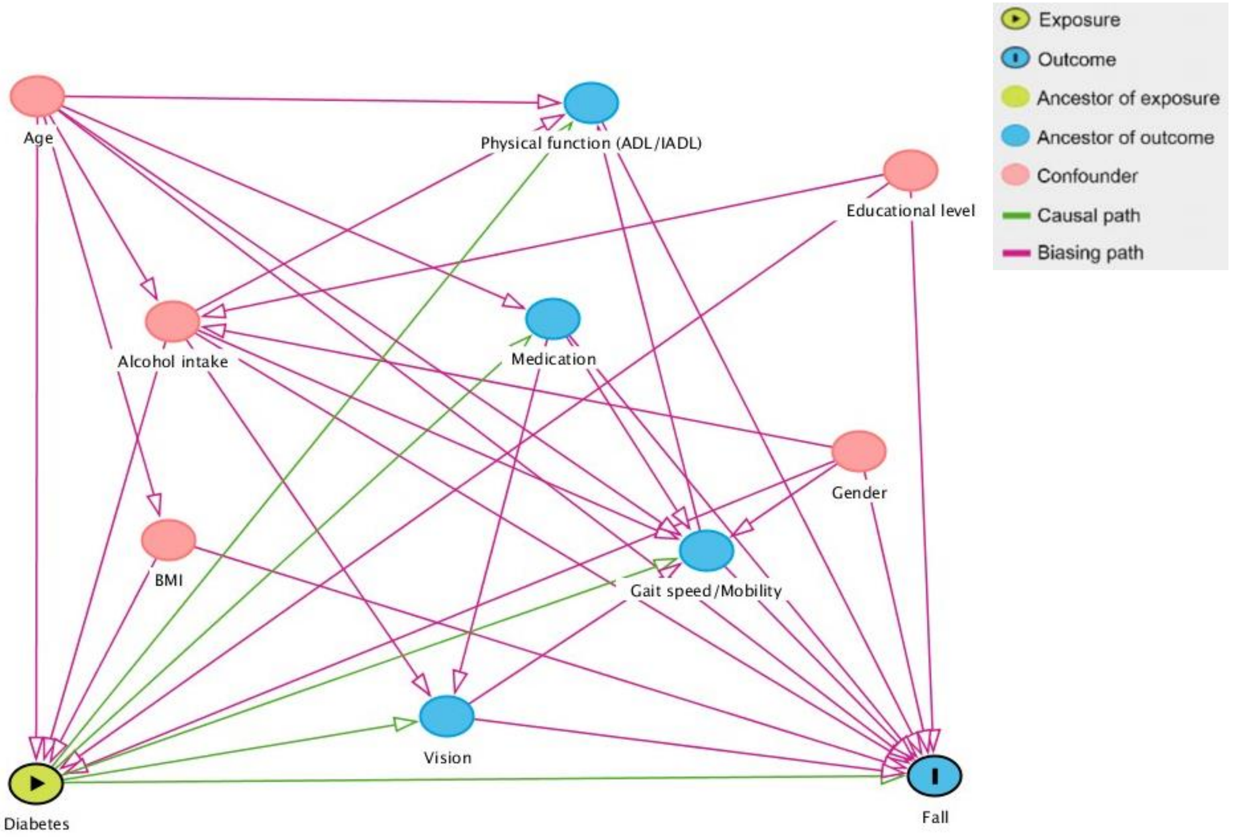

Fig 5S. Directed Acyclic Graphs for causal inference framework of diabetes to fall among older outpatient. Minimal Sufficient Adjustment Sets: age, gender, educational level, BMI and alcohol intake. The graph was drawn from https://www.dagitty.net

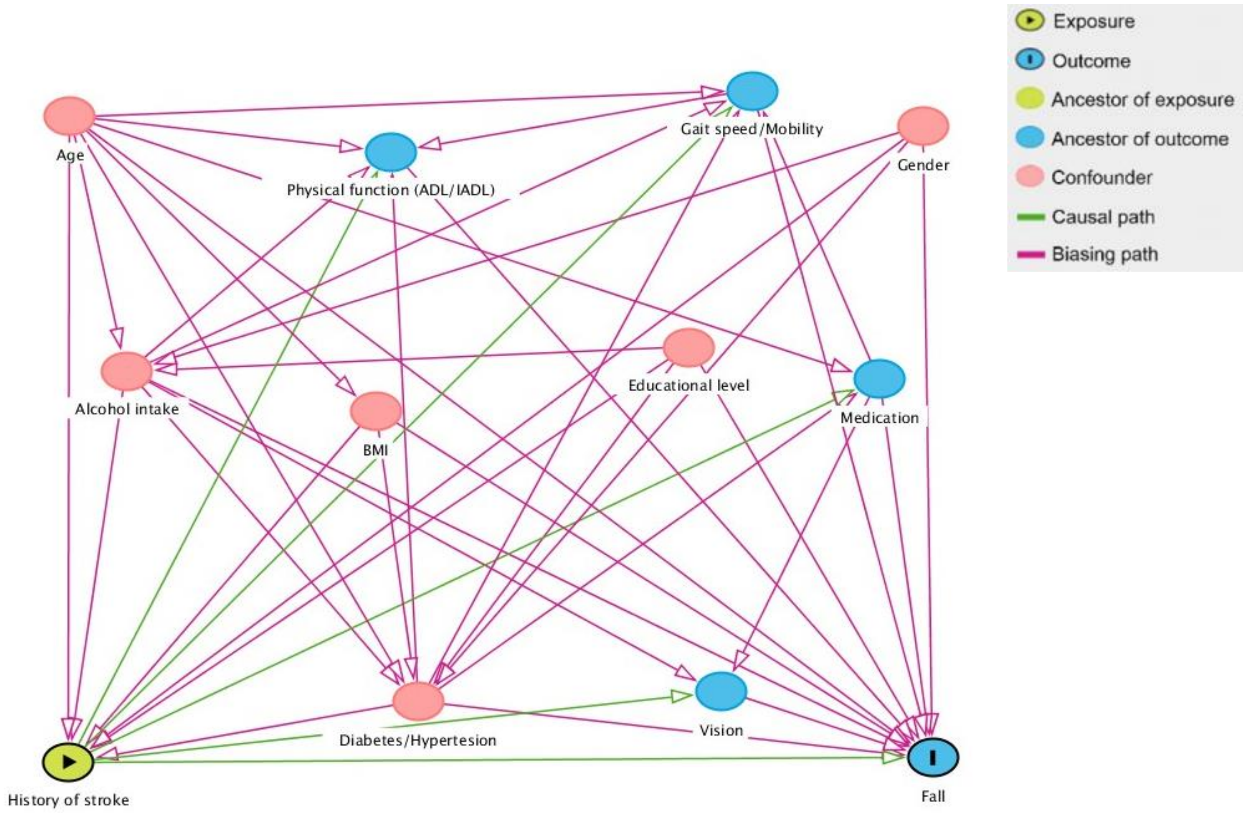

Fig 6S. Directed Acyclic Graphs for causal inference framework of history of stroke to fall among older outpatient. Minimal Sufficient Adjustment Sets: age, gender, educational level, BMI, alcohol intake, diabetes and hypertension. The graph was drawn from https://www.dagitty.net

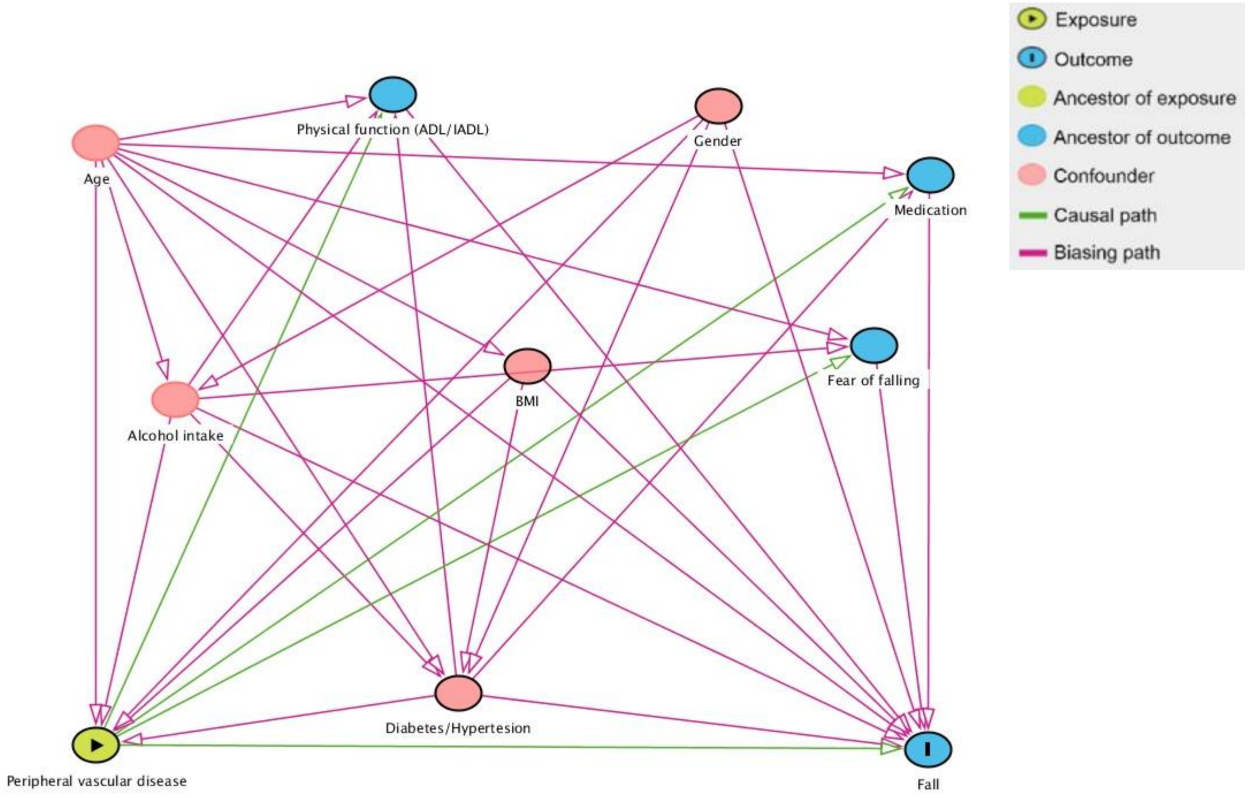

Fig 7S. Directed Acyclic Graphs for causal inference framework of peripheral vascular disease to fall among older outpatient. Minimal Sufficient Adjustment Sets: age, gender, BMI, alcohol intake, diabetes and hypertension. The graph was drawn from https://www.dagitty.net

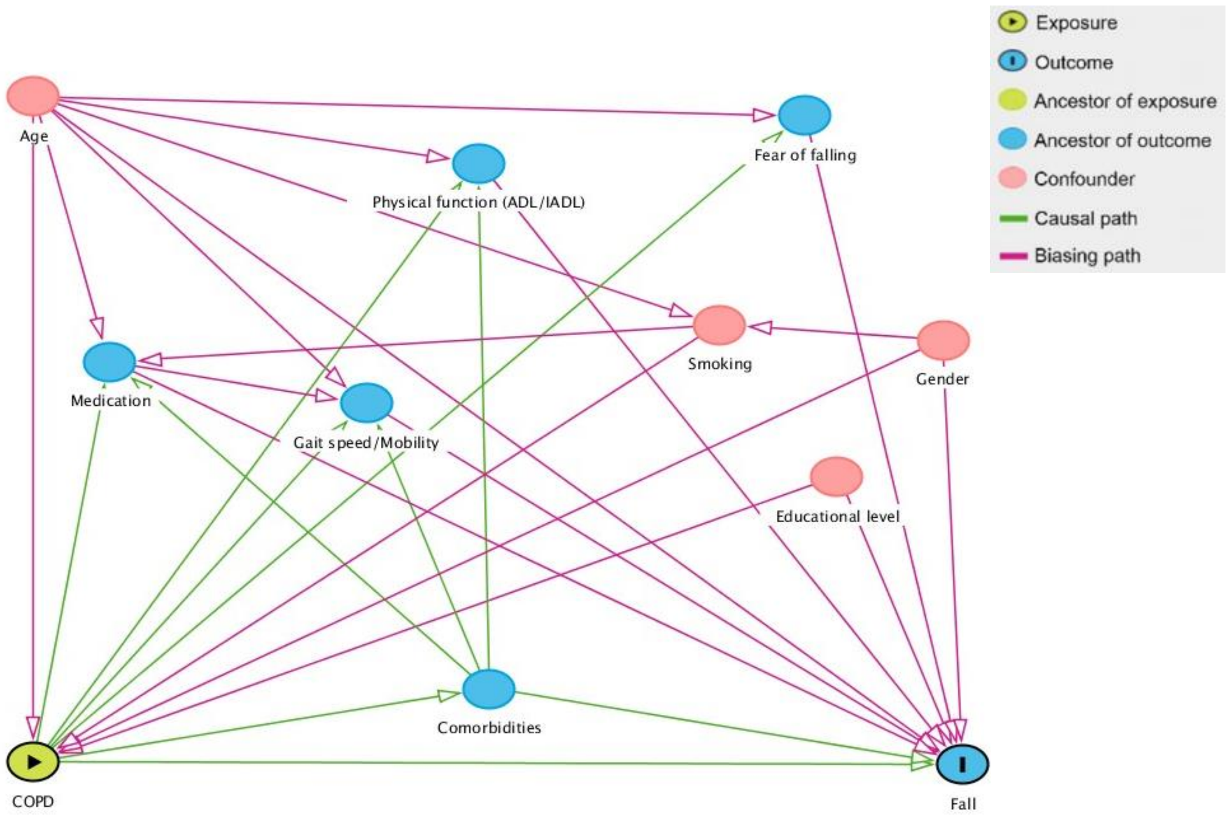

Fig 8S. Directed Acyclic Graphs for causal inference framework of COPD to fall among older outpatient. Minimal Sufficient Adjustment Sets: age, gender, educational level and smoking. The graph was drawn from https://www.dagitty.net

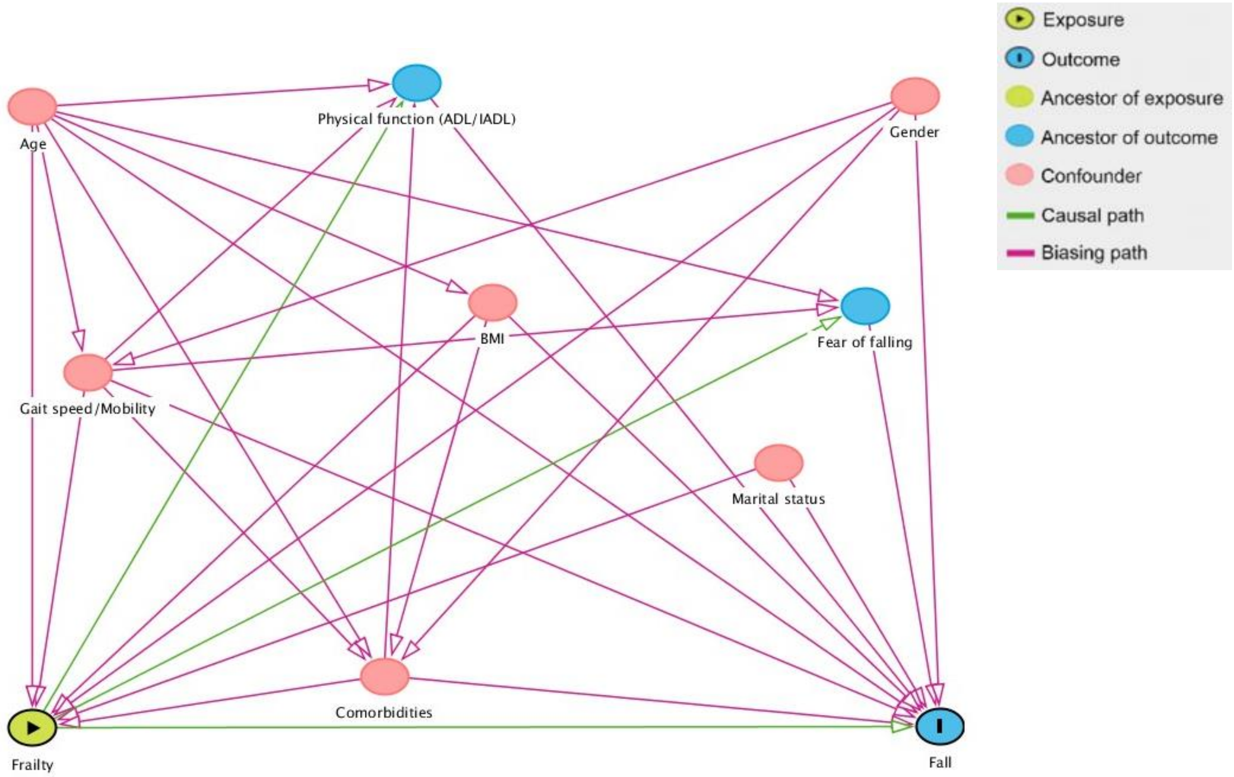

Fig 9S. Directed Acyclic Graphs for causal inference framework of frailty to fall among older outpatient. Minimal Sufficient Adjustment Sets: age, gender, BMI, marital status, comorbidities, gait speed and mobility. The graph was drawn from https://www.dagitty.net

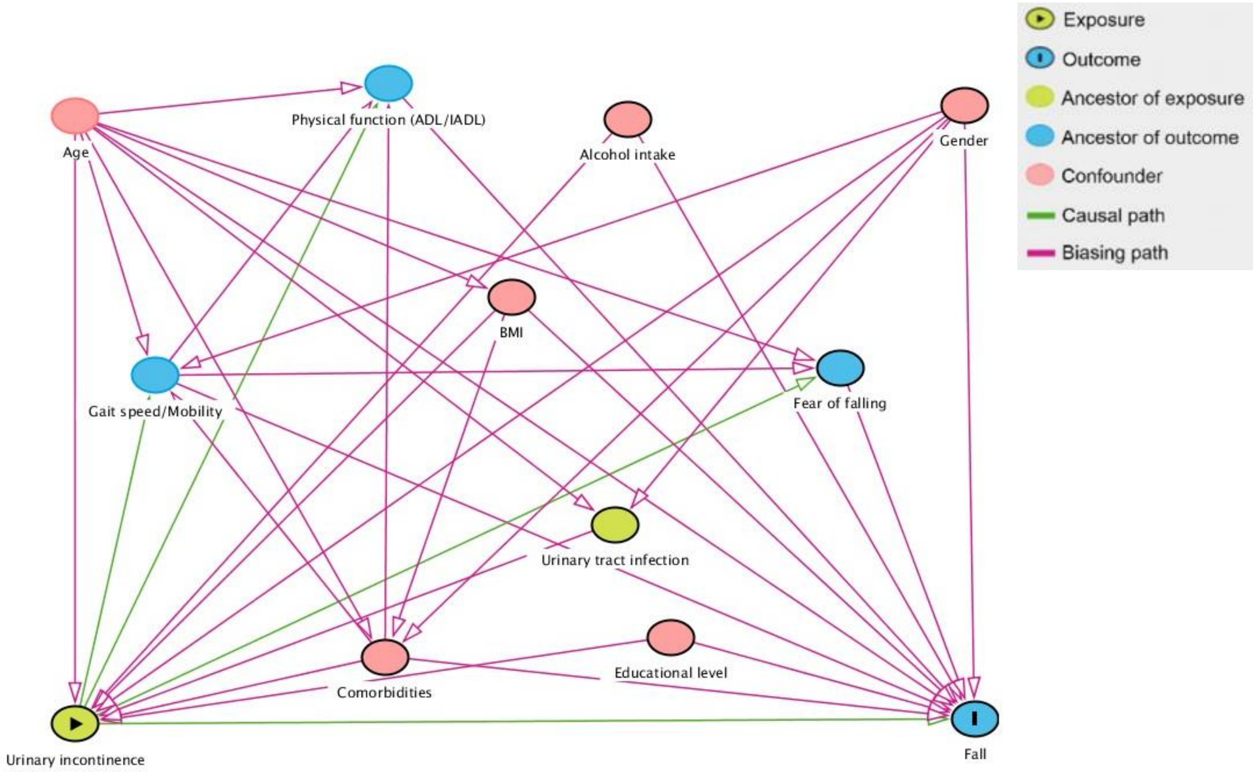

Fig 10S. Directed Acyclic Graphs for causal inference framework of urinary incontinence to fall among older outpatient. Minimal Sufficient Adjustment Sets: age, gender, BMI, educational level, comorbidities and alcohol intake. The graph was drawn from https://www.dagitty.net

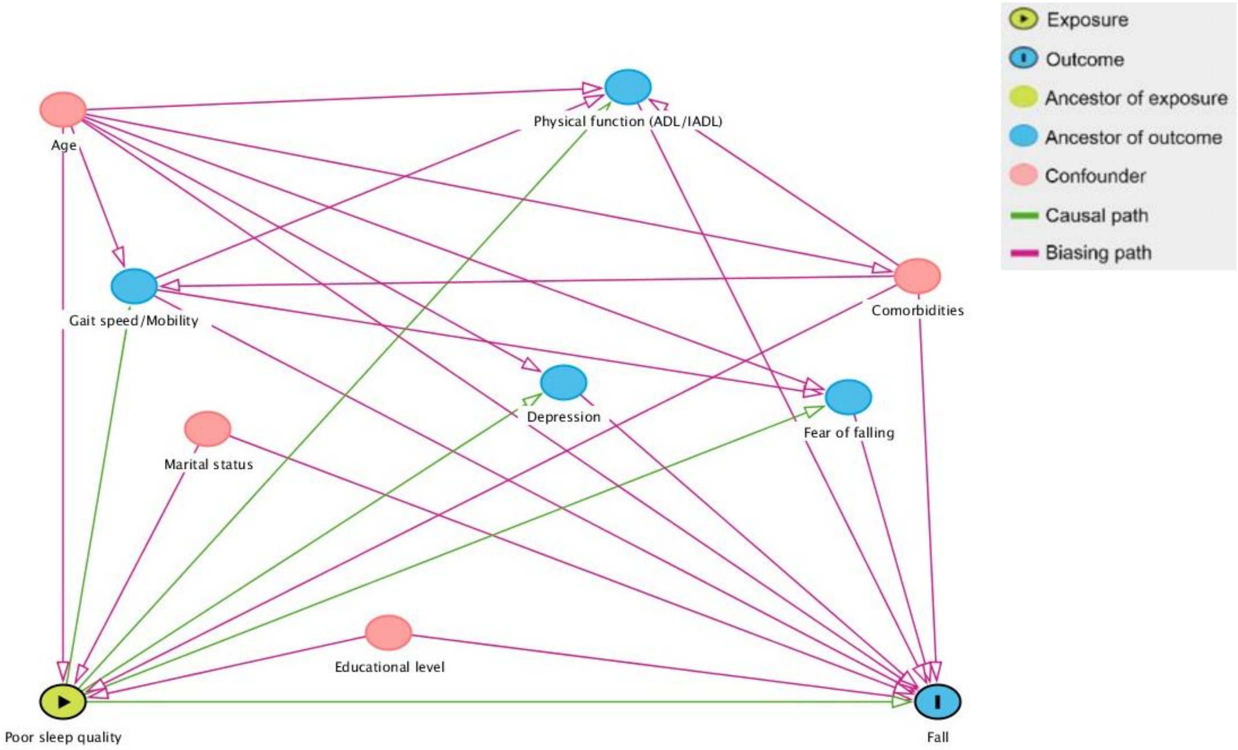

Fig 11S. Directed Acyclic Graphs for causal inference framework of poor sleep quality to fall among older outpatient. Minimal Sufficient Adjustment Sets: age, educational level, comorbidities, marital status. The graph was drawn from https://www.dagitty.net

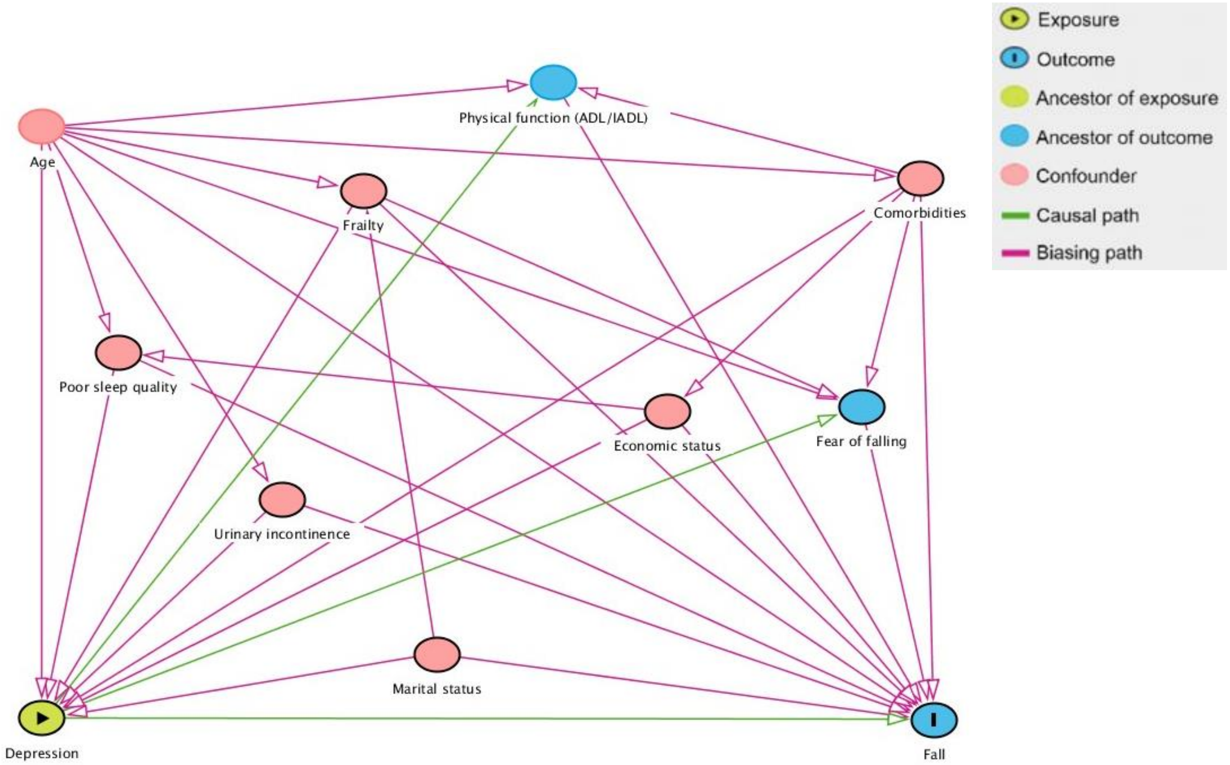

Fig 12S. Directed Acyclic Graphs for causal inference framework of depression to fall among older outpatient. Minimal Sufficient Adjustment Sets: age, comorbidities, marital status, poor sleep quality, economic status, frailty and urinary incontinence. The graph was drawn from https://www.dagitty.net

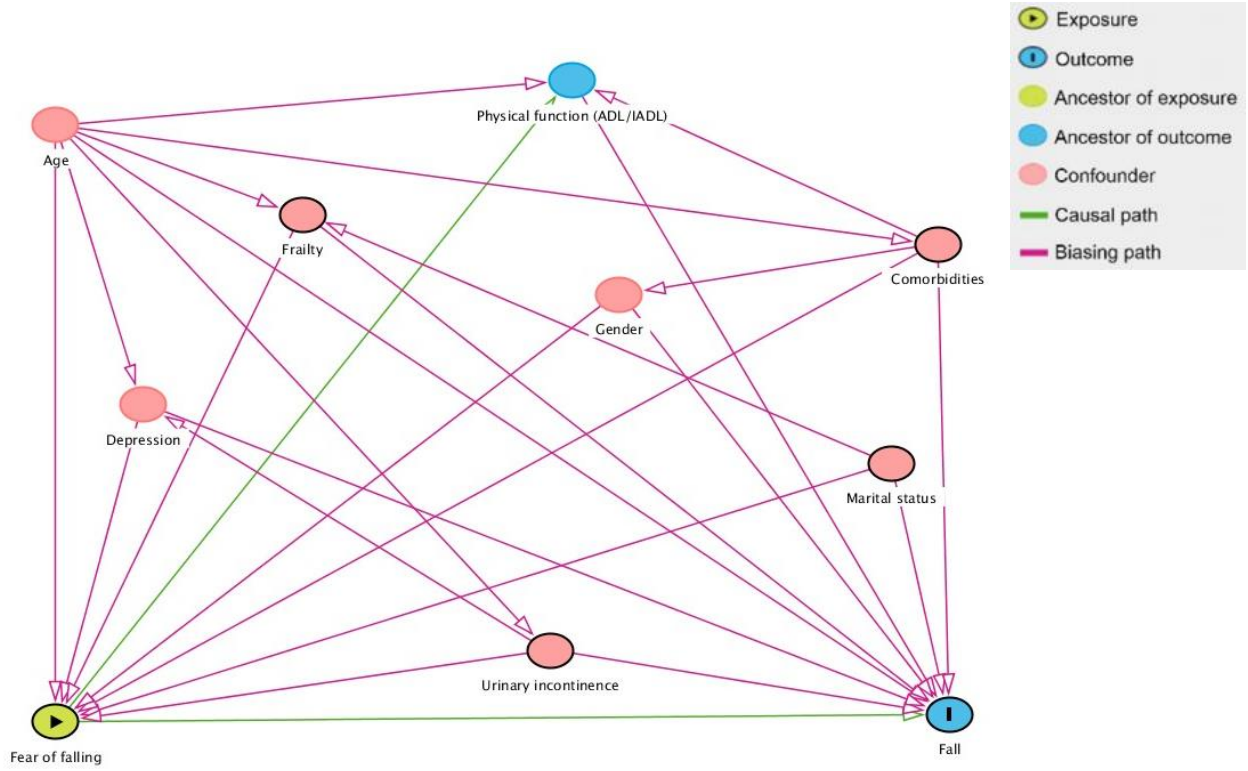

Fig 13S. Directed Acyclic Graphs for causal inference framework of fear of falling to fall among older outpatient. Minimal Sufficient Adjustment Sets: age, gender, comorbidities, marital status, frailty, urinary incontinence and depression. The graph was drawn from https://www.dagitty.net

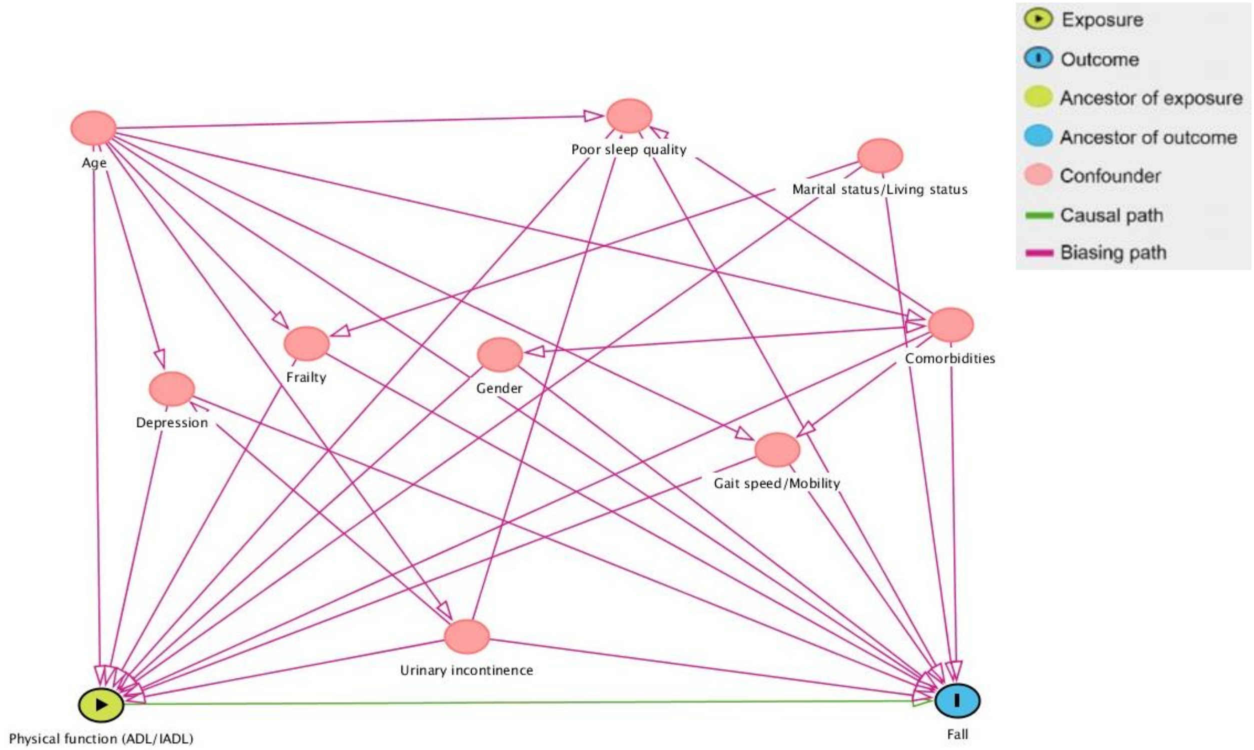

Fig 14S. Directed Acyclic Graphs for causal inference framework of physical function (ADL/IADL) to fall among older outpatient. Minimal Sufficient Adjustment Sets: age, gender, comorbidities, marital status, frailty, urinary incontinence, depression, gait speed, mobility and poor sleep quality. The graph was drawn from https://www.dagitty.net

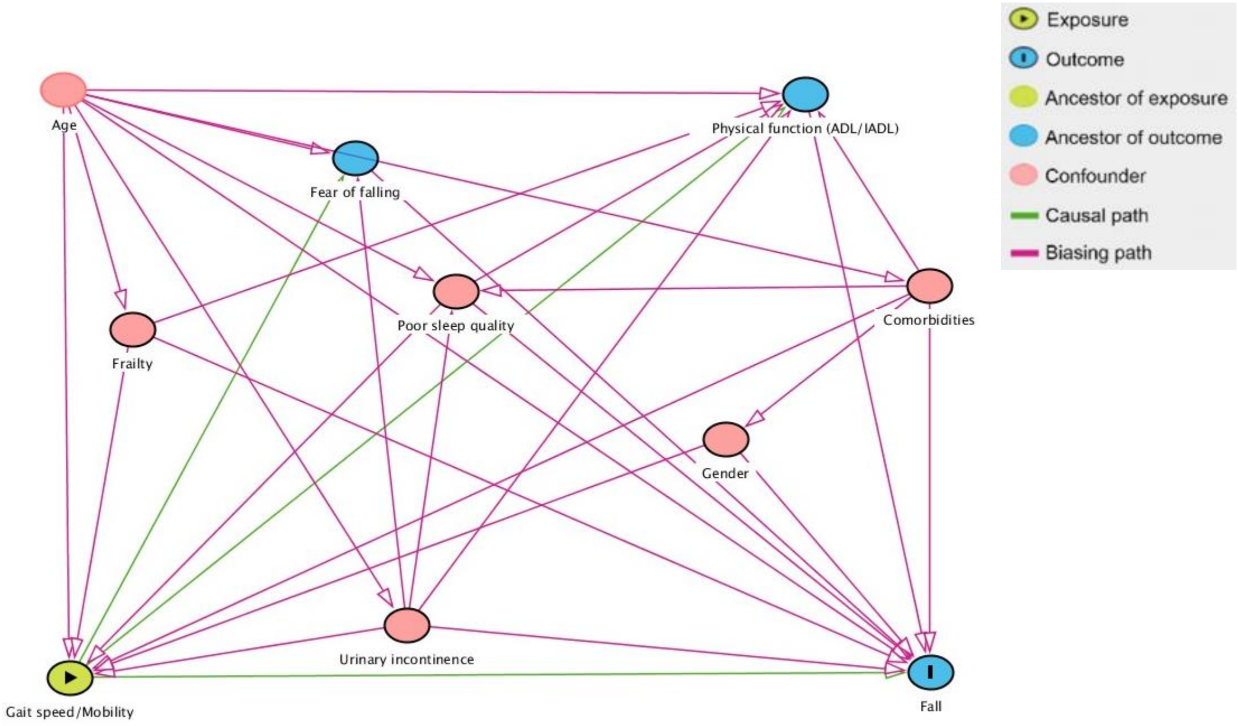

Fig 15S. Directed Acyclic Graphs for causal inference framework of physical function (ADL/IADL) to fall among older outpatient. Minimal Sufficient Adjustment Sets: age, gender, comorbidities, frailty, urinary incontinence and poor sleep quality. The graph was drawn from https://www.dagitty.net
